# Neuromuscular junction-specific genes screening by deep RNA-seq analysis

**DOI:** 10.1186/s13578-021-00590-9

**Published:** 2021-05-01

**Authors:** Tiankun Hui, Hongyang Jing, Xinsheng Lai

**Affiliations:** 1 School of Life Science, Nanchang University, Nanchang, Jiangxi China; 2 Laboratory of Synaptic Development and Plasticity, Institute of Life Science, Nanchang University, Nanchang, Jiangxi China

**Keywords:** Neuromuscular junction, RNA-seq, Differentially expressed genes, NMJ diseases

## Abstract

**Background:**

Neuromuscular junctions (NMJs) are chemical synapses formed between motor neurons and skeletal muscle fibers and are essential for controlling muscle contraction. NMJ dysfunction causes motor disorders, muscle wasting, and even breathing difficulties. Increasing evidence suggests that many NMJ disorders are closely related to alterations in specific gene products that are highly concentrated in the synaptic region of the muscle. However, many of these proteins are still undiscovered. Thus, screening for NMJ-specific proteins is essential for studying NMJ and the pathogenesis of NMJ diseases.

**Results:**

In this study, synaptic regions (SRs) and nonsynaptic regions (NSRs) of diaphragm samples from newborn (P0) and adult (3-month-old) mice were used for RNA-seq. A total of 92 and 182 genes were identified as differentially expressed between the SR and NSR in newborn and adult mice, respectively. Meanwhile, a total of 1563 genes were identified as differentially expressed between the newborn SR and adult SR. Gene Ontology (GO) enrichment analyses, Kyoto Encyclopedia of Genes and Genomes (KEGG) analysis and gene set enrichment analysis (GSEA) of the DEGs were performed. Protein–protein interaction (PPI) networks were constructed using STRING and Cytoscape. Further analysis identified some novel proteins and pathways that may be important for NMJ development, maintenance and maturation. Specifically, Sv2b, Ptgir, Gabrb3, P2rx3, Dlgap1 and Rims1 may play roles in NMJ development. Hcn1 may localize to the muscle membrane to regulate NMJ maintenance. Trim63, Fbxo32 and several Asb family proteins may regulate muscle developmental-related processes.

**Conclusion:**

Here, we present a complete dataset describing the spatiotemporal transcriptome changes in synaptic genes and important synaptic pathways. The neuronal projection-related pathway, ion channel activity and neuroactive ligand-receptor interaction pathway are important for NMJ development. The myelination and voltage-gated ion channel activity pathway may be important for NMJ maintenance. These data will facilitate the understanding of the molecular mechanisms underlying the development and maintenance of NMJ and the pathogenesis of NMJ disorders.

## Background

Synapses are fundamental units of neural circuitry that are responsible for communications between multiple neurons or between neurons and their targets. As important synapses that form between motoneurons and skeletal muscle fibers, neuromuscular junctions (NMJs) are essential for transducing motor neuron signals to muscle and initiating skeletal muscle contraction. During the past decade, some proteins that are critical for NMJ formation have been identified, such as Agrin, Lpr4, MuSK, Dok7, and Rapsyn [1–8]. In NMJs, neural Agrin binds Lrp4, a transmembrane protein of the LDL receptor family, to activate the receptor tyrosine kinase MuSK [3, 4]. The downstream of Musk are not well understood except for Rapsyn, which is reported to exhibit E3 ligase activity and is required for agrin-induced acetylcholine receptor (AChR) clustering [9]. In addition, recent studies have indicated that these core proteins are critical for NMJ maintenance. Loss of Agrin, Lrp4 or Dok7 after NMJ formation leads to NMJ disintegration and dysfunction [10–12].

NMJ impairment results in many diseases, such as congenital myasthenic syndrome (CMS) and myasthenia gravis (MG) [13, 14]. MG is one of the most common acquired autoimmune disorders of the NMJ, affecting 400–600 per 1,000,000 individuals. It can be diagnosed by identifying antibodies against the AChR and MuSK [15, 16]. Recently, antibodies against Agrin and Lrp4 have been identified in MG patients [17–22]. CMS usually has an early onset and is less common than MG. Protein mutations in CMS can be classified as in presynaptic, synaptic, basal lamina-associated, or postsynaptic regions. Mutations in ChAT (Choline acetyltransferase) are associated with presynaptic defects in CMS, and mutations in ColQ and LAMB2 cause synaptic defects in CMS. Mutations in key genes of the NMJ, including *Agrin*, *Lrp4*, *MuSK*, *Dok7*, *AChR subunits* and *Rapsyn*, are all associated with the pathogenesis of CMS [13].

These findings suggest that the specific regulation of gene expression in NMJs during NMJ development and maintenance affects disease progression. However, in many patients, the genetic cause of the disease is still unclear. Thus, identification of these NMJ-specific proteins is an important step. Because skeletal muscles are multinucleated cells and NMJs represent only 0.01–0.1% of muscle fibers, some nuclei are synaptic, whereas the majority are not synaptic. Moreover, many of the critical proteins are extremely concentrated at NMJs. Thus, identifying NMJ-specific proteins will provide us with information to reveal novel mechanisms of NMJ development, maintenance and even NMJ-related diseases. Although previous studies have attempted to screen for synaptic genes that are critical for NMJ formation, only a limited number of genes have been identified, and comprehensive comparison of the transcriptome of synaptic genes at different ages is lacking [23, 24].

Herein, we aimed to identify novel proteins in NMJs that are important for NMJ development, maintenance and maturation. Synaptic and nonsynaptic regions of the diaphragm muscles were isolated from mice of different ages and subjected to RNA-seq. Subsequently, GO enrichment analysis, KEGG analysis, GSEA and PPI network analyses were performed. We found that the neuron projection pathway and neuroactive ligand-receptor interaction pathway are important for NMJ development. The myelination and voltage-gated ion channel activity pathways are important for NMJ maintenance. Together, these observations uncover the pathways that are important for NMJ development, maintenance and disease.

## Materials and methods

### RNA extraction and library construction

The synaptic region and nonsynaptic region of NMJs were dissected from the diaphragm under a stereomicroscope. Samples were separated and placed in RNA-free PBS. RNA was extracted using TRIzol (Sigma, T9424) according to the manufacturer’s protocol. After concentration measurement, the integrity of RNA was assessed by the Agilent 2100 Bioanalyzer system. The libraries for RNA-seq were constructed with the TruSeq RNA Sample Prep Kit (Illumina, San Diego, CA, USA) according to the manufacturer’s instructions.

### RNA-seq data processing and DEG identification

Raw reads were subjected to quality control. Clean reads were filtered and mapped to mouse reference genome assembly using TopHat 2.0.13 [25]. Gene expression levels were quantified by Cufflinks 2.2.1 and normalized by the fragments per kilobase of transcript per million fragments mapped method (FPKM). After standardization, the DEGs were identified by EdgeR package. We compared the genes of the postnatal day 0 (P0) mouse synaptic region with the corresponding nonsynaptic region, adult mouse synaptic region with the corresponding nonsynaptic region, and adult mouse synaptic region with the P0 mouse synaptic region and considered these DEGs to be genes critical for NMJ development, maintenance and maturation. The DEGs were selected with p-value < 0.05 and log_2_FC (fold change) > 2 and log_2_FC < − 2.

### Immunofluorescence

Muscles were fixed in 4% paraformaldehyde (PFA) at room temperature for 30 min. Next, the samples were incubated with blocking buffer (2% BSA, 7% goat serum and 0.5% Triton X-100 in PBS) for 2–4 h at room temperature. After washing 3 times for 15 min each, the samples were incubated with neurofilament (CST, C28E10, 1:1000) and synapsin (CST, D12G5, 1:1000) antibodies overnight at 4 °C. The tissues were then washed 3 times and incubated with AlexaFluor-488 goat anti-rabbit IgG (Invitrogen, A-11034, 1:1000) secondary antibody and CF568 α-bungarotoxin (α-BTX, Biotium, #00006, 1:3000) for 2–4 h at room temperature. After washing 3 times for 30 min in PBS, tissues were flat-mounted with Hydromount (National Diagnostics).

### DEG pathway analyses

We performed GO analysis by Database for Annotation, Visualization and Integrated Discovery (DAVID) [26]. GSEA was applied to identify the a priori-defined gene sets that showed statistically significant differences between synaptic regions and nonsynaptic regions of P0 and adult mice. We performed this analysis using an online database (https://www.omicstudio.cn/index).

### PPI network construction and module analysis

The PPI network was predicted using the STRING online database [27]. STRING provides insights into the proteins associated with these DEGs. In this study, an interaction with a combined score > 0.4 was considered statistically significant. The PPI network was drawn with Cytoscape. The most significant module in the PPI network was identified by the MCODE plugin in Cytoscape.

### Quantitative real-time PCR

Total RNA was isolated from the synaptic region and nonsynaptic region of the diaphragm using TRIzol reagent. RNA was reverse transcribed into cDNA with a High Capacity cDNA Reverse Transcription kit (4368814, Applied Biosystems). cDNA was used as a template for Q-PCR, and RT-PCR was performed with SYBR Green qPCR Master Mix (100029284, Takara). Loading standards were as previously described, and each sample was measured in triplicate. mRNA levels were normalized to GAPDH mRNA levels. The primers for specific genes are listed in Table 1.

**Table 1:** Primer sequence used for qRT-PCR

Primer	Forward	Reverse
*GAPDH*	CAT CAC TGC CAC CCA GAA GAC TG	ATG CCA GTG AGC TTC CCG TTC AG
*Sv2b*	GCA GAC TCA TCT CAG GCA TAG G	CCT CCA GTC ATC CAG AAG ATG C
*Osbp2*	ATC ACC ATC GCC AGC AAG TTC C	AGG TGC TCT TCC TCC ACA CGT A
*Scrg1*	AGA GCC GAC CTG AAG CTG ATA G	ACA AAG GAG ATC TTT GGT CCA AAG
*Scg5*	GGA CTT CAG TGA GGA TCA AGG C	GGA ATT CTC GGC TGA ACT CTG C
*Map6d1*	CGG ACG GTC TAC GTG CTG CC	GAG TTG TGA CGA CTC TGG CTG T
*Pla2g5*	CTG TCA GAT GCA CGA CCG TTG T	GAG CCT CAT TGG ACA GAA GGA G
*Elavl4*	ACC TCA CGC ATC CTG GTT GAT C	GTA ATC GGT TCT GTA GCA CCG C
*P2rx3*	TCA TCA ACC GAG CCG TTC AGC T	ACT CTG TTG GCA TAG CGT CCG A
*Slc6a11*	GGT TTG CCA TCT TCT CAG TCC TG	GGG CAT CAT AGT GAC AGC CTT G
*Hcn1*	CTA TGA GCA CCG ATA CCA AGG C	GGC ATA GTA GCC ACC AGT TTC C
*Ccl21c*	TCC CTA CAG TAT TGT CCG AGG C	ATC AGG TTC TGC ACC CAG CCT T
*Pou3f1*	ACA GCC TGC AAC TGG AGA AGG A	CAG GCG CAT AAA CGT CGT CCA T
*Ajap1*	TAG CAC AAC GGA GCC TTC CAC T	TGA TGA GGG AGA CGG TGA TGG T
*Lphn3*	CTC TTG CAG AGC CTA TGT CCA G	CAG TGT CAA GCA ACA TGG TGG C
*Ddn*	CTC CTT CTC GAC AGT CCA TGG A	GTT GCC TCC TGC AGC ACC CTG
*Etv4*	CAC AGA CTT CGC CTA CGA CTC A	CAC AGA CTT CGC CTA CGA CTC A
*Sox10*	TCT ACT TCT GCT TGC CGC TAG C	CAA ACA CGA GGA CCA GGC AGA A
*Cxcl14*	TAC CCA CAC TGC GAG GAG AAG A	CGC TTC TCG TTC CAG GCA TTG T
*Clic6*	GAT GGT GAA GTC AAG ACA GAT GTG	CAT TTC CCG CTG AGT TGG ACT C
*Apod*	GGT GAA GCC AAA CAG AGC AAC G	CAG GAG TAC ACG AGG GCA TAG T
*Actr3b*	AAG AGT GGT GGA CGC CAG GTT A	AGC CTC CAA ACC ACA CGG CAT A
*Adig*	CCG TGG CTT TGC TGC TGT TCT T	CTC AGA TGG TCT TTT GCT CCA GG
*BC048679*	GTT TAC TGC GGC CAA GGA GAG A	CAC AGT TGC TGC TAG TGG GAT G
*Lrrc52*	CCT GGA TAT GCA ACT GCT CCT TC	AGT GGA TTC CCC ACC TTC GTG A
*Zdhhc23*	GGA TAT GCG GTA TCT GTG TAC GG	GGT CAG CGA TAT TCC GTA AAC CG

## Results

### Analysis of synaptic and nonsynaptic regions at P0 revealed pathways for NMJ development

**Fig. 1:**
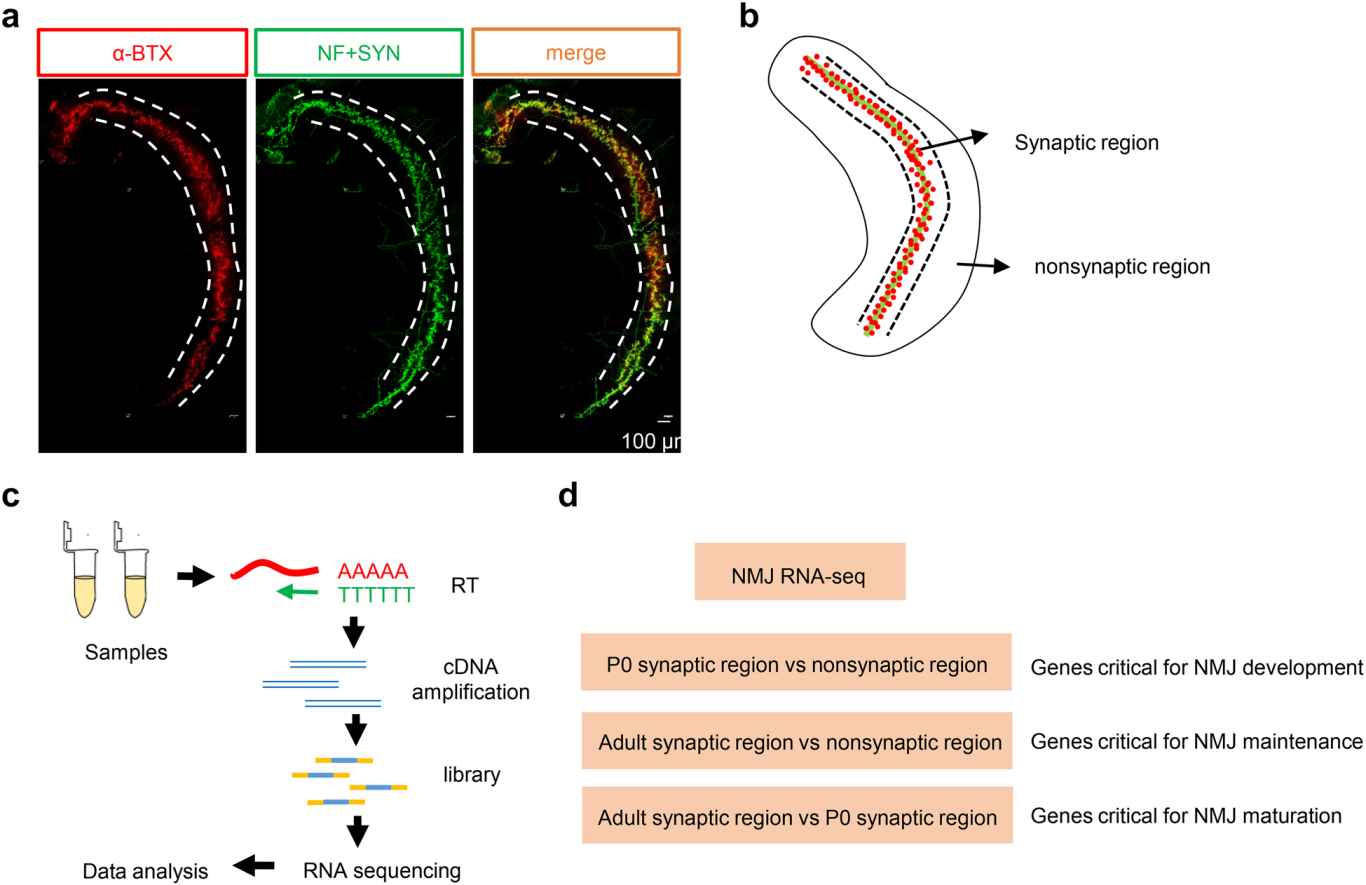
Overview of the screening strategy.** a** Representative image of a P0 diaphragm. Muscles were stained with CF568 α-BTX (red) and anti-NF/SYN (green) antibodies. **b** Diagram of the NMJ synaptic region and nonsynaptic region. Green indicates the primary nerve, and red indicates AChRs. **c** The NMJ RNA-seq procedure. **d** Three pairs of comparisons

To investigate the molecular mechanisms underlying the structural and functional changes during NMJ maturation and further identify the candidate molecules that regulate this process and NMJ-related diseases, we performed RNA-seq on muscles from P0 and adult mice. As shown in Fig. 1a, the diaphragms of P0 mice were characterized to visualize NMJs with neurofilament and synapsin (NF/SYN) and α-BTX staining. In the diaphragm, AChR clusters were concentrated in the middle area, which was named the “synaptic region”, and the side area where no AChR clusters were present was named the “nonsynaptic” region (Fig. 1b). The general workflow of NMJ RNA-seq was shown in Fig. 1c. Here, we analyzed three different groups: P0 synaptic region vs nonsynaptic region, adult synaptic region vs nonsynaptic region, and adult synaptic region vs P0 synaptic region (Fig. 1d).

**Fig. 2:**
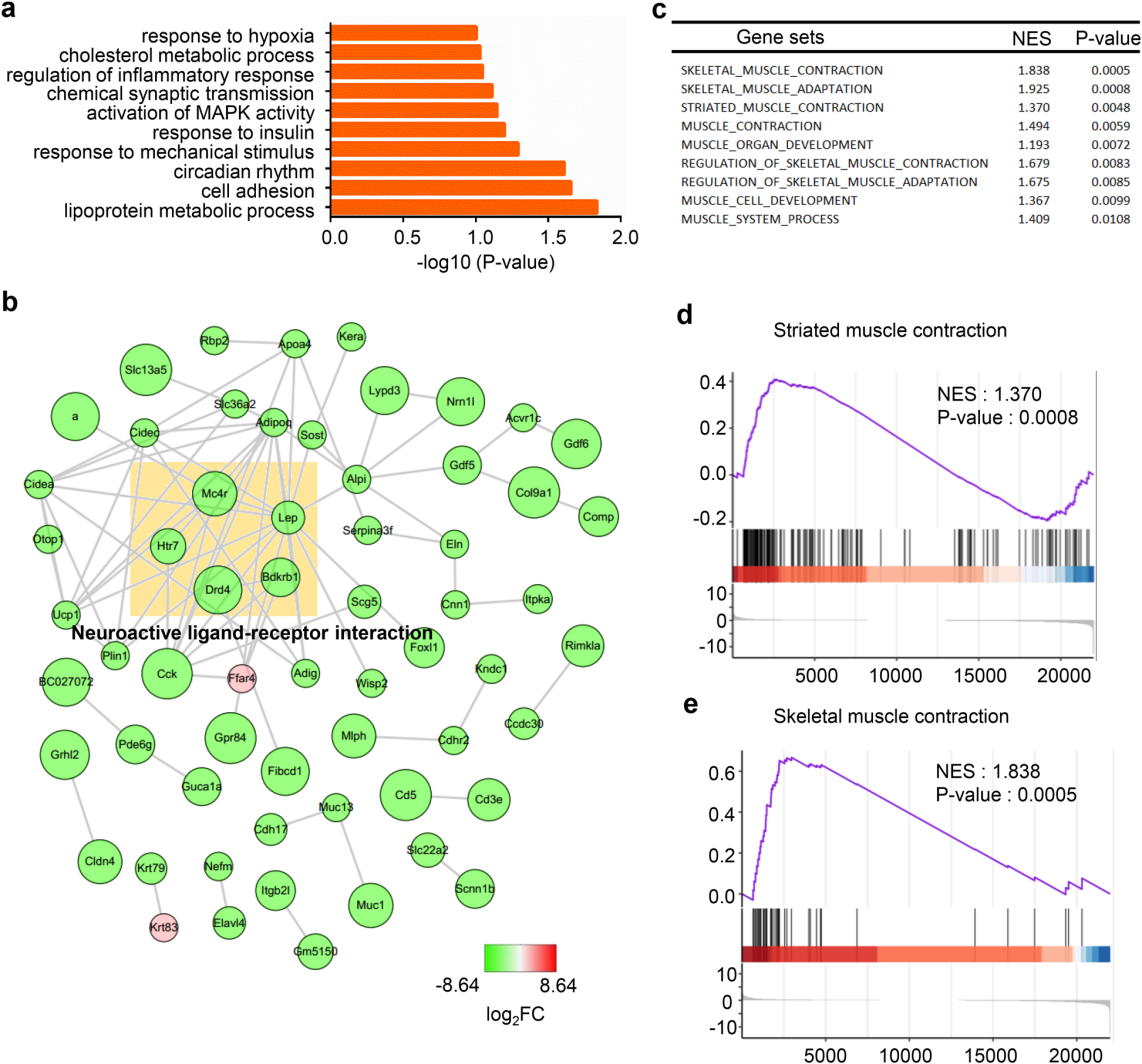
Analysis of DEGs critical for NMJ development.** a** GO enrichment analysis of the DEGs. **b** The PPI network of the DEGs was constructed by Cytoscape. The color depth of the nodes indicates the log_2_FC, and the size of nodes indicates the p-value of the ontologies. *Mc4r*, *Lep*, *Htr7*, *Drd4* and *Bdkrb1* in this network were found within the neuroactive ligand-receptor interaction pathway. **c** Selected gene sets related to the NMJ. **d**, **e** Selected enriched gene sets from the GSEA revealed that the genes in striated muscle contraction and skeletal muscle contraction gene sets are active in the P0 synaptic region compared with the P0 nonsynaptic region. The purple trace shows the enrichment score

To identify genes that are critical for NMJ development, the synaptic and nonsynaptic regions of the diaphragm from P0 mice were analyzed. In total, 92 DEGs were found; 16 genes were upregulated, while 76 genes were downregulated. To understand the functions of DEGs, biological process analysis was performed by DAVID. We found that the DEGs were mainly enriched in chemical synaptic transmission, cell adhesion and response to mechanical stimulus pathways (Fig. 2a). Additionally, the PPI network of the DEGs was drawn using Cytoscape. These DEGs may cooperate to achieve the biological functions corresponding to this network. We analyzed the gene functions that may be related to NMJ and found that the genes in the light yellow box are related to neuroactive ligand-receptor interactions (Fig. 2b). Since NMJs consist of muscle, neurons and Schwann cells, we focused on the DEG functions enriched among these components. Next, we performed GSEA and found that skeletal muscle contraction and developmental-related gene sets were more active in the synaptic region than in the nonsynaptic region at P0 (Fig. 2c–e).

**Fig. 3:**
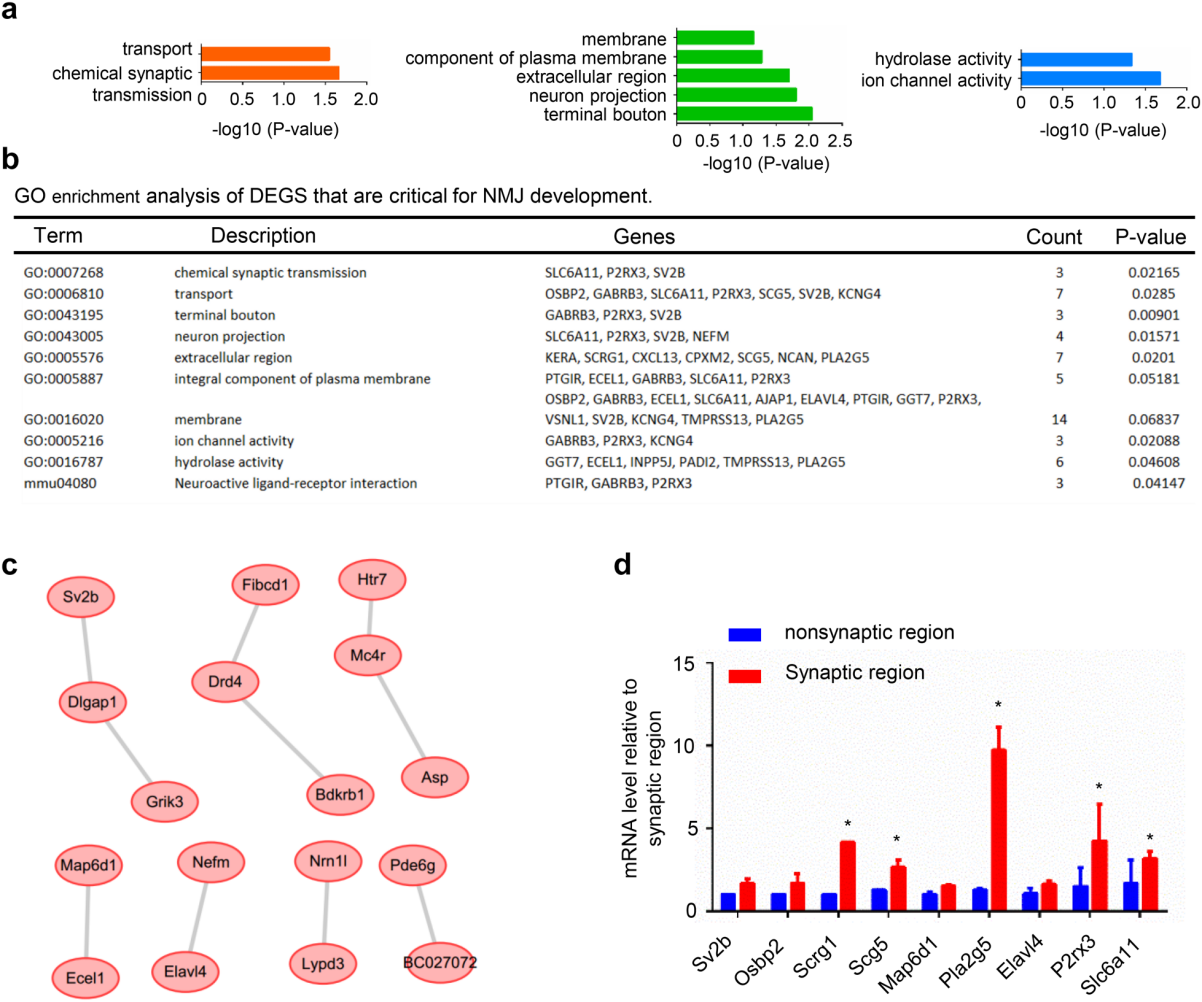
Analysis of highly expressed genes that are critical for NMJ development. **a** BP, CC and MF analysis of highly expressed genes. **b** GO enrichment analysis of highly expressed genes that are critical for NMJ development. **c** PPI network construction of highly expressed genes. **d** Q-PCR detection of some DEGs and hub genes

We next focused on the highly expressed genes of synaptic region (p-value < 0.05 and log_2_Foldchange > 2) among the DEGs. The GO analysis showed that the biological processes (BPs) of these genes were significantly enriched in chemical synaptic transmission and transport. The main enriched cellular components (CCs) were terminal boutons, neuron projections and extracellular regions. The enriched molecular functions (MFs) were mainly ion channel activity and hydrolase activity (Fig. 3a). KEGG pathway analysis showed enrichment in the neuroactive ligand-receptor interaction pathway (Fig. 3b). From the GO results, we found that Sv2b, which is located in terminal boutons, was involved in chemical synaptic transmission, transport and neuron projection. We thus deduced from this result that Sv2b may be located in the motor nerve terminal to regulate NMJ transmission and motor neuron projection. The neuroactive ligand-receptor interaction pathway proteins Ptgir, Gabrb3 and P2rx3 may be related to the NMJ AChR receptor. However, these findings need further exploration. In addition, a PPI network was constructed (Fig. 3c). Among the identified genes, Sv2b was found to be essential for regulating neurotransmitter release [28]. To validate the DEGs identified by RNA-seq, the mRNA levels of some DEGs and hub genes were measured by quantitative real-time PCR. Consistent with the RNA-seq results, many of the detected genes were upregulated at the synaptic region of muscles from P0 mice (Fig. 3d). These results provide us with a reference to study NMJ development and related diseases.

### Analysis of adult mouse synaptic and nonsynaptic regions revealed pathways for NMJ maintenance

**Fig. 4:**
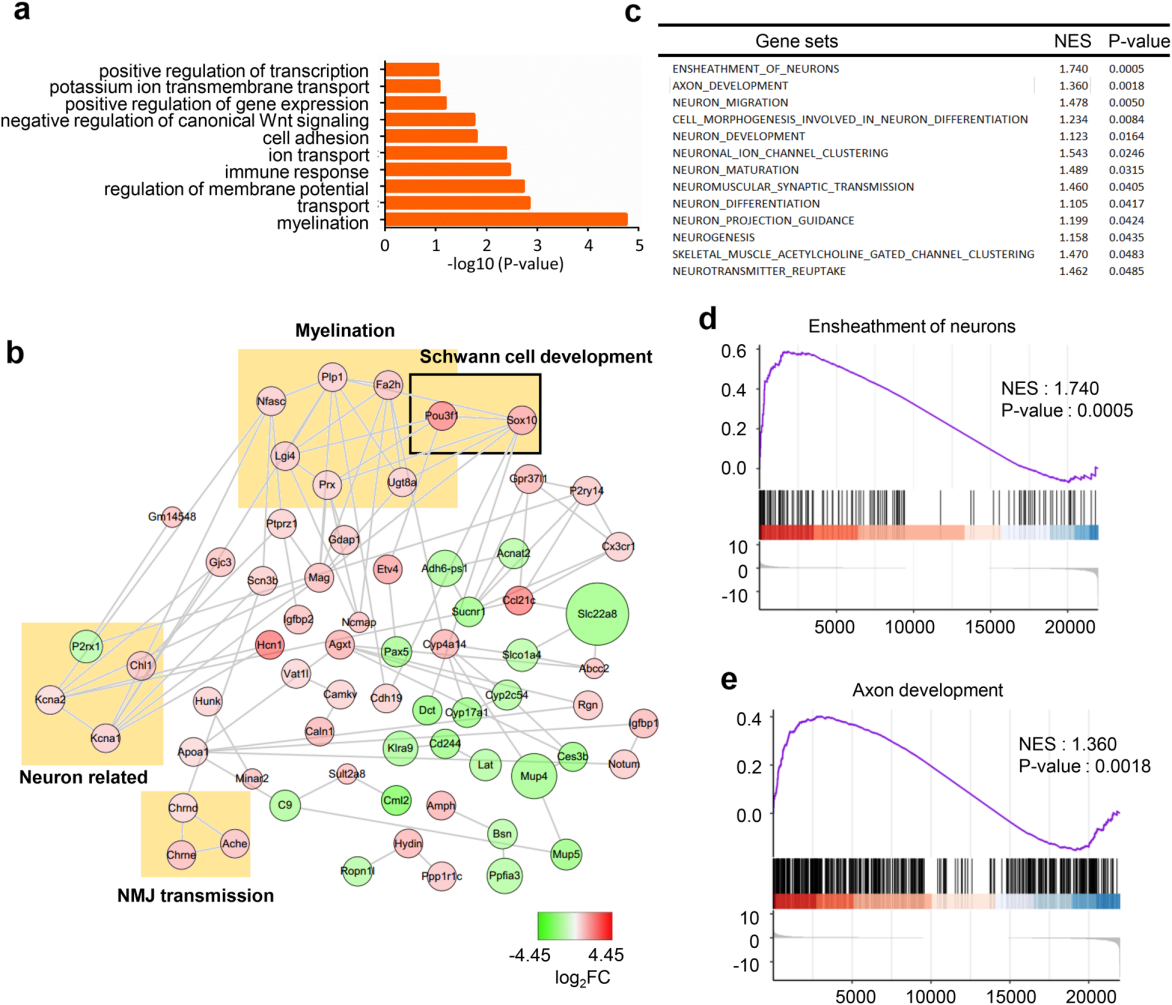
Analysis of DEGs that are critical for NMJ maintenance. **a** The DEGs are enriched in myelination and transport pathways. **b** A PPI network was constructed by using Cytoscape. Some genes were found to have relationships with myelination, Schwann cell development, neurons, and NMJ transmission. **c** Selected gene sets related to the NMJ. **d**, **e** Selected enriched gene sets from the GSEA revealed that genes in the ensheathment of neurons and axon development gene sets are active in the adult synaptic region compared with the adult nonsynaptic region

To identify genes that are critical for the maintenance of NMJs, the synaptic and nonsynaptic regions of the diaphragm from 3-month-old mice were analyzed. In total, 182 DEGs were found; 34 genes were upregulated in the synaptic regions, while 148 genes were downregulated. GO analysis revealed that DEGs were enriched in myelination, transport and regulation of membrane potential pathways (Fig. 4a). Many of the genes in the PPI network were found to have relationships with myelination, Schwann cell development, neuron-related and NMJ transmission pathways (Fig. 4b). In addition, we found that ensheathment of axon, axon development and many other neuron-related gene sets were more active in the synaptic region than in the nonsynaptic region by GSEA (Fig. 4c–e).

**Fig. 5:**
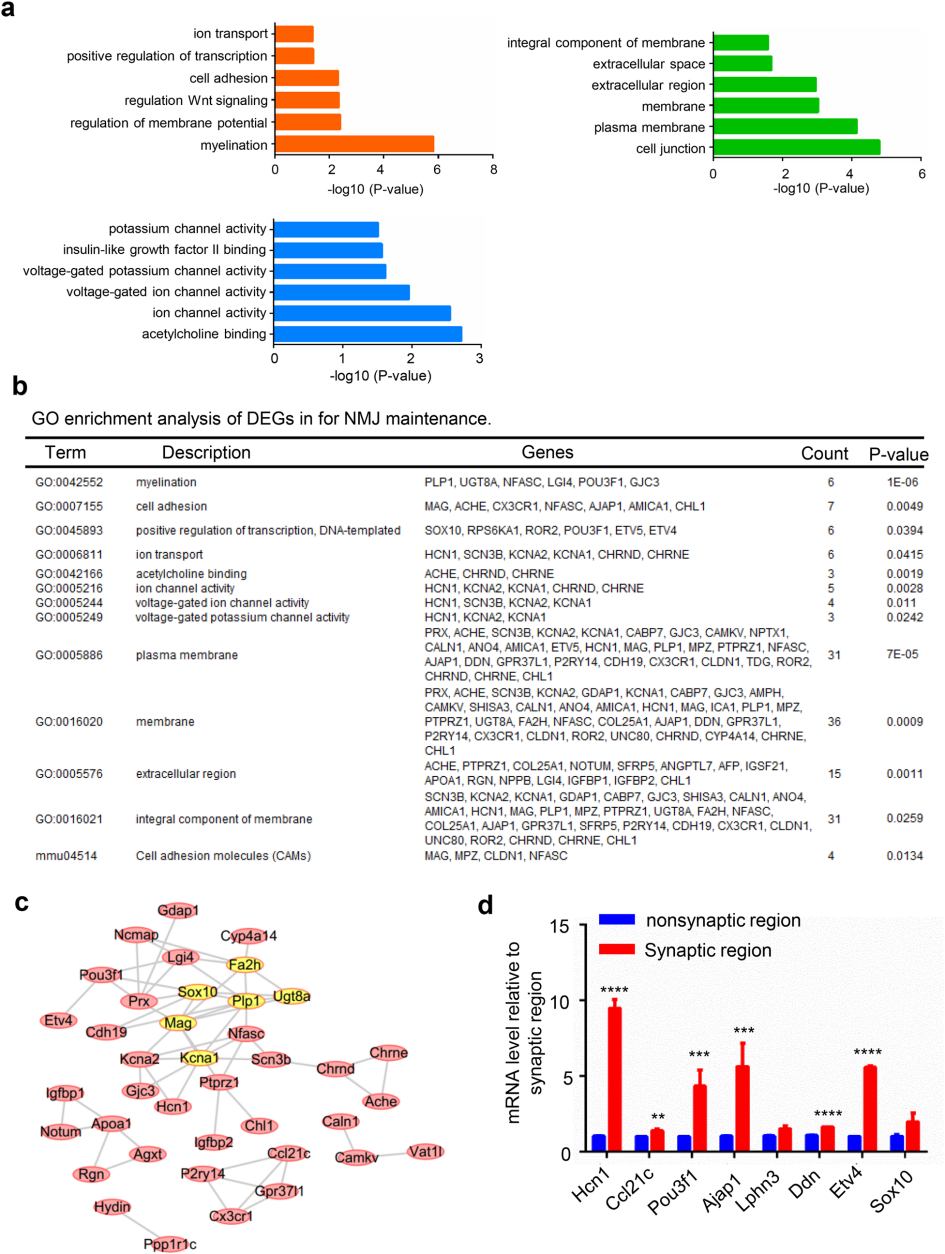
Analysis of highly expressed genes that are critical for NMJ maintenance.** a** BP, CC and MF analysis of highly expressed genes in adult mice. **b** GO enrichment and KEGG pathway analysis of genes that are critical for NMJ maintenance. **c** Constructed PPI network of highly expressed genes. The hub genes in this network were highly related to myelination. **d** Q-PCR detection of some DEGs and hub genes revealed that these genes are highly expressed in the NMJs of adult mice

Next, we performed GO analysis of the genes that were highly expressed in the synapses. We found that DEGs were enriched in myelination, regulation of membrane potential and cell adhesion processes. The CCs of DEGs were mainly enriched in plasma membrane, cell junction and membrane. The MFs were mainly enriched in acetylcholine binding, ion channel activity and voltage-gated ion channel activity (Fig. 5a). KEGG analysis showed that the target genes were enriched in cell adhesion molecules (Fig. 5b). It is conceivable that the DEGs are enriched in the myelination process because the sample includes axons and the associated myelinated Schwann cells. Among these DEGs, Hcn1 was reported to be predominantly expressed in the brain to regulate some brain disorders, such as epilepsy and posttraumatic stress disorders [29]. In this result, Hcn1 was mainly located in the membrane to regulate voltage-gated ion channel activity. Combined with the function of AChRs, which are voltage-gated Na^+^ channels in NMJs that trigger action potentials [30], we deduced that Hcn1 may be located in the muscle membrane and combine with Na^+^ channels to regulate electrical activity in NMJs. In addition, the PPI network was constructed using the STRING database, and the most significant module was drawn. Most of the six hub genes (*Mag*, *Ugt8a*, *Plp1*, *Kcna1*, *Sox10* and *Fa2h*) with degrees ≥ 10 were related to the myelination process (Fig. 5c). The names, abbreviations, and functions of these hub genes are shown in Table 2. We next validated some DEGs and hub genes by using quantitative real-time PCR and found that the mRNA levels of* Hcn1*, *Ccl21c*, *Pou3f1*, *Ajap1*, *Ddn*, *Etv4*, and *Sox10* were identically upregulated, which was consistent with the RNA-seq results (Fig. 5d).

**Table 2:** Hub genes with degree ≥ 10 in DEGs between adult SR and NSR

Gene	Full name	Function
*Mag*	Guanylate-binding protein 1	Hydrolyzes GTP to GMP in 2 consecutive cleavage reactions. Exhibits antiviral activity against influenza virus (By similarity). Promote oxidative killing and deliver antimicrobial peptides to autophagolysosomes, providing broad host protection against different pathogen classes
*Ugt8a*	2-Hydroxyacylsphingosine 1-beta-galactosyltransferase	Catalyzes the transfer of galactose to ceramide, a key enzymatic step in the biosynthesis of galactocerebrosides, which are abundant sphingolipids of the myelin membrane of the central nervous system and peripheral nervous system
*Plp1*	Myelin proteolipid protein	This is the major myelin protein from the central nervous system. It plays an important role in the formation or maintenance of the multilamellar structure of myelin
*Kcna1*	Potassium voltage-gated channel subfamily A member 1	Contributes to the regulation of the membrane potential and nerve signaling, and prevents neuronal hyperexcitability
*Sox10*	Transcription factor SOX-10	Transcription factor that plays a central role in developing and mature glia. Specifically activates expression of myelin genes, during oligodendrocyte (OL) maturation, such as DUSP15 and MYRF, thereby playing a central role in oligodendrocyte maturation and CNS myelination
*Fa2h*	Fatty acid 2-hydroxylase	Catalyzes stereospecific hydroxylation of free fatty acids at the C-2 position to produce (*R*)-2-hydroxy fatty acids, which are building blocks of sphingolipids and glycosphingolipids common in neural tissue and epidermis

### Analysis of the adult mouse synaptic region and P0 mouse synaptic region revealed pathways for NMJ maturation

**Fig. 6:**
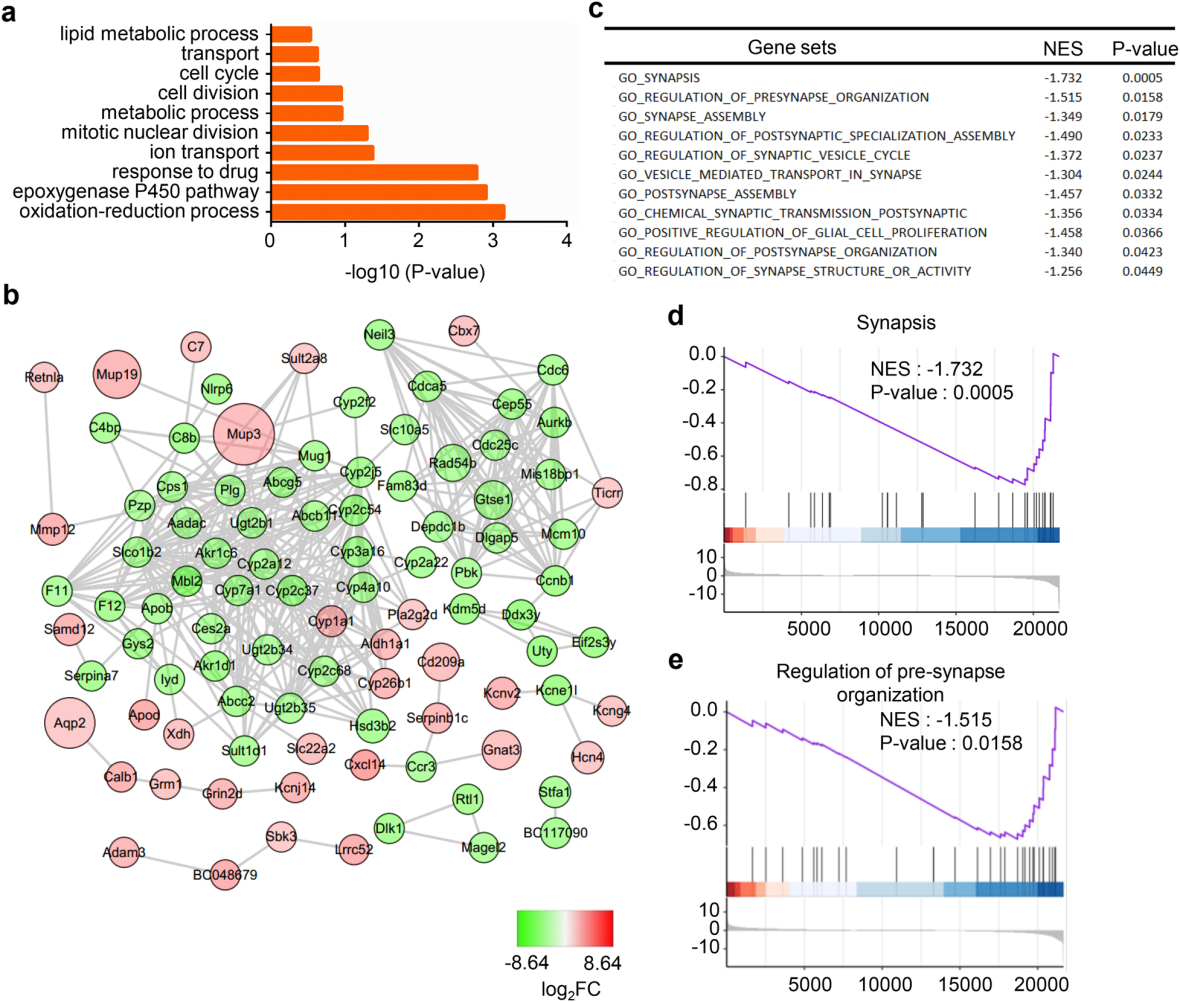
Analysis of DEGs that are critical for NMJ maturation. **a** The DEGs were enriched in the oxidation–reduction process and ion transport pathways. **b** The PPI network of the DEGs was constructed by Cytoscape. **c** Selected gene sets related to the NMJ. **d**, **e** Selected enriched gene sets from the GSEA revealed that the genes in the synapsis and regulation of presynapse organization gene sets are inactive in adult mice compared with P0 mice

**Fig. 7:**
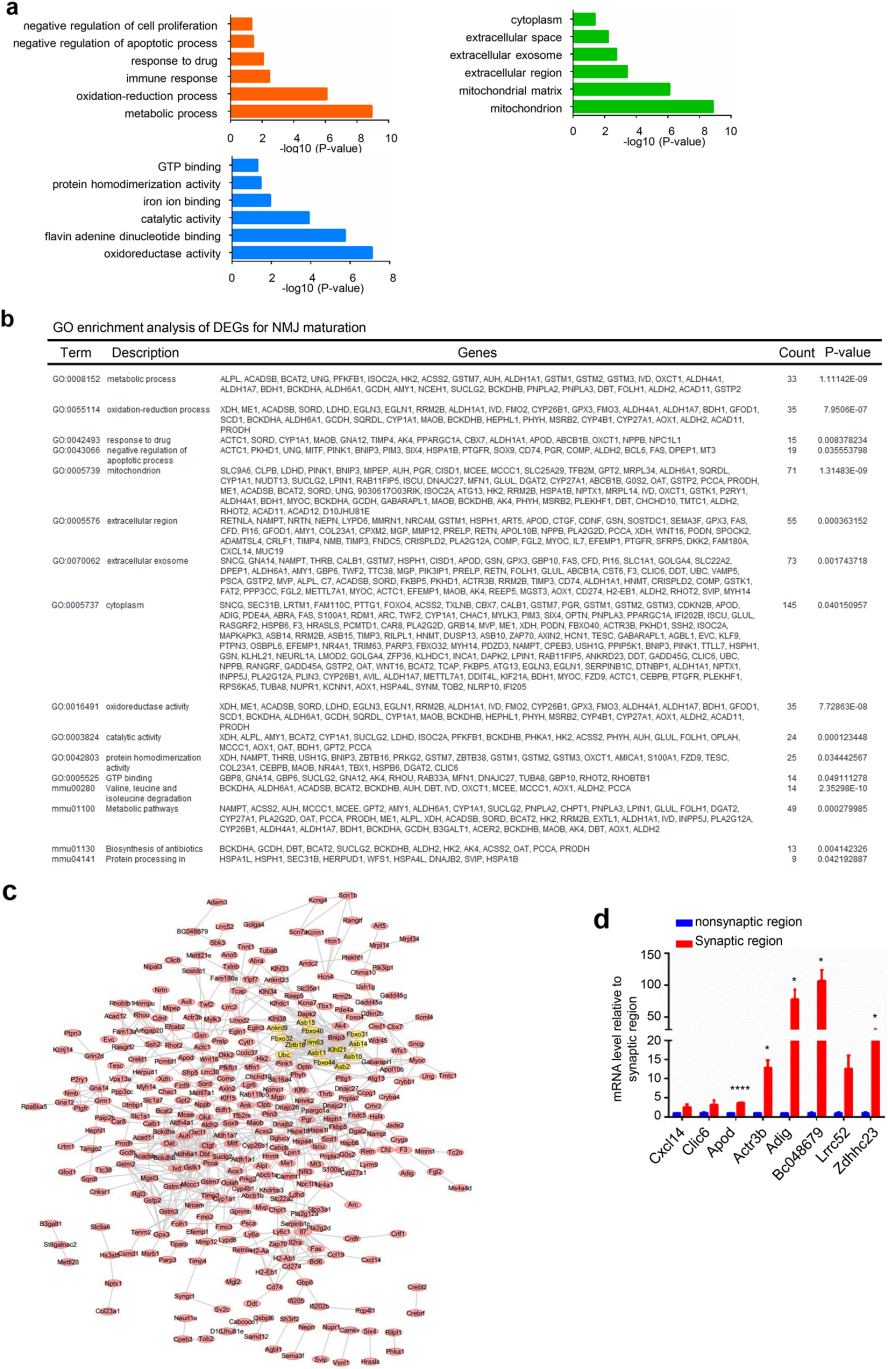
Analysis of highly expressed genes critical for NMJ maturation. **a** BP, CC and MF analysis of highly expressed genes by adult synaptic region and P0 synaptic region comparison. **b** GO enrichment and KEGG pathway analysis of genes that are critical for NMJ maturation. **c** The PPI network of highly expressed genes was constructed by Cytoscape. Many Asb family proteins were identified as hub genes. **d** Q-PCR detection of some DEGs and hub genes revealed that these genes were highly expressed in the NMJs of adult mice compared with those of P0 mice

For NMJ maturation analysis, a total of 1563 DEGs were found. Among these genes, 363 were upregulated and 1200 were downregulated. By analyzing the synaptic regions of adult and P0 mice, we found that the DEGs were significantly enriched in the oxidation–reduction process, response to drug and ion transport pathways (Fig. 6a). A PPI network was constructed, and the relationships of the DEGs are displayed (Fig. 6b). By GSEA, we found that synapsis and regulation of synapse organization gene sets were highly active in the P0 mouse synaptic region compared with the adult mouse synaptic region, which confirmed that developmental-related pathways were active in P0 mice (Fig. 6c–e). To further understand the functions of DEGs that are critical for NMJ maturation, we performed GO analysis of the significantly upregulated genes. Metabolic processes and immune responses were found to be enriched in adult mouse synaptic regions. The enriched CCs were mitochondria, mitochondrial matrix and extracellular region. The enriched MFs were oxidoreductase activity, catalytic activity and protein homodimerization activity (Fig. 7a, b). Q-PCR data confirmed the significant DEGs and hub genes that were highly expressed in the adult mouse synaptic region (Fig. 7c). By PPI network construction, 14 hub genes were identified with degrees ≥ 10, including *Ankrd9*, *Klhl21*, *Asb11*, *Fbxo44*, *Fbxo32*, *Trim63*, *Zbtb16*, *Fbxo31*, *Asb14*, *Ubc*, *Fbxo40*, *Asb2* and *Asb10* (Fig. 7d). Obviously, many Asb family proteins were enriched in this module. Asb family proteins have been reported to have high expression in muscle. The names, abbreviations, and functions of these hub genes are shown in Table 3.

**Table 3:** Hub genes with degree ≥ 10 in DEGs between adult SR and P0 SR

Gene	Full name	Function
*Zbtb16*	Zinc finger and BTB domain containing 16	DNA-binding transcription repressor activity, RNA polymerase II-specific
*Ubc*	SUMO-conjugating enzyme UBC9	Accepts the ubiquitin-like proteins SUMO1, SUMO2 and SUMO3 from the UBLE1A-UBLE1B E1 complex and catalyzes their covalent attachment to other proteins with the help of an E3 ligase
*Asb14*	Ankyrin repeat and SOCS box protein 14	May be a substrate-recognition component of a SCF-like ECS E3 ubiquitin-protein ligase complex which mediates the ubiquitination and subsequent proteasomal degradation of target proteins
*Fbxo44*	F-box only protein 44	Substrate-recognition component of the SCF -type E3 ubiquitin ligase complex
*Asb11*	Ankyrin repeat and SOCS box protein 11	May be a substrate-recognition component of a SCF-like ECS (Elongin-Cullin-SOCS-box protein) E3 ubiquitin-protein ligase complex which mediates the ubiquitination and subsequent proteasomal degradation of target proteins
*Asb2*	Ankyrin repeat and SOCS box protein 2	Probable substrate-recognition component of a SCF-like ECS (Elongin-Cullin-SOCS-box protein) E3 ubiquitin-protein ligase complex which mediates the ubiquitination and subsequent proteasomal degradation of target proteins
*Klhl21*	Kelch-like protein 21	Substrate-specific adapter of a BCR (BTB-CUL3-RBX1) E3 ubiquitin-protein ligase complex required for efficient chromosome alignment and cytokinesis
*Fbxo44*	F-box only protein 44	Substrate-recognition component of the SCF (SKP1-CUL1-F-box protein)-type E3 ubiquitin ligase complex
*Asb10*	Ankyrin repeat and SOCS box protein 10	May be a substrate-recognition component of a SCF-like ECS E3 ubiquitin-protein ligase complex which mediates the ubiquitination and subsequent proteasomal degradation of target proteins
*Asb15*	Ankyrin repeat and SOCS box protein 15	May be a substrate-recognition component of a SCF-like ECS (Elongin-Cullin-SOCS-box protein) E3 ubiquitin-protein ligase complex which mediates the ubiquitination and subsequent proteasomal degradation of target proteins
*Trim63*	E3 ubiquitin-protein ligase TRIM63	E3 ubiquitin ligase. Mediates the ubiquitination and subsequent proteasomal degradation of CKM, GMEB1 and HIBADH. Regulates the proteasomal degradation of muscle proteins under amino acid starvation, where muscle protein is catabolized to provide other organs with amino acids
*Fbxo32*	F-box only protein 32	Substrate recognition component of a SCF (SKP1-CUL1-F-box protein) E3 ubiquitin-protein ligase complex which mediates the ubiquitination and subsequent proteasomal degradation of target proteins

### Irregular NMJ function-related pathways were not enriched in the synaptic region of NMJ development and maintenance

**Fig. 8:**
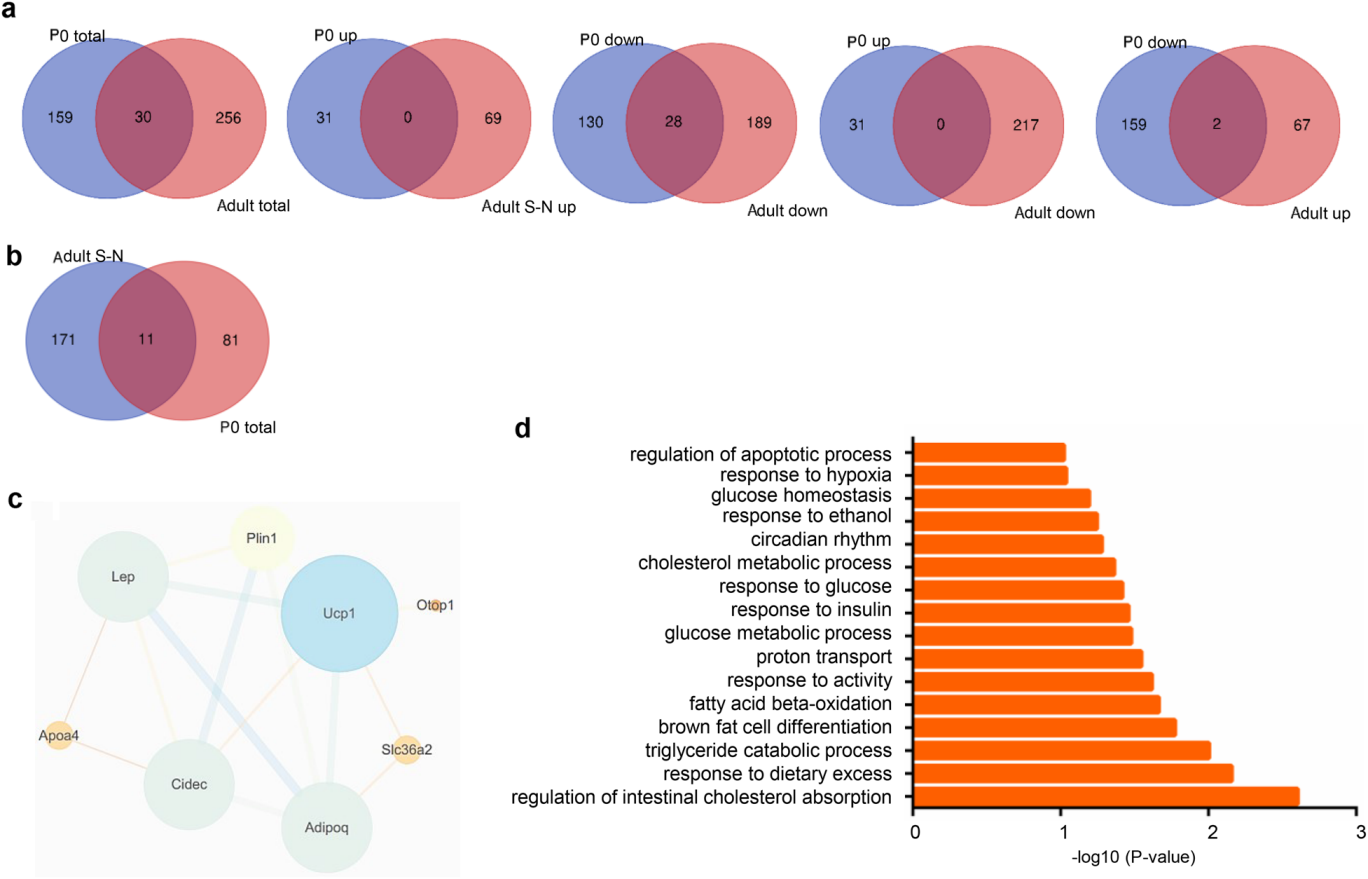
The downregulated genes in the synaptic region were not enriched in regular NMJ function-related pathways. **a** Venn diagrams of DEGs with -1.5 < log_2_FC > 1.5 in NMJs of P0 and adults were drawn. **b** Venn diagrams of DEGs with -2 > log_2_FC > 2 in NMJs of P0 and adults were drawn, and 11 genes were found. **c** The PPI network of the 11 genes was constructed, and 8 genes had relationships with each other. **d** The GO enrichment analysis of the 11 genes showed that these genes were not enriched in regular NMJ function-related pathways

Next, we drew a Venn diagram of the highly expressed synaptic genes in P0 and adult mice. First, we screened the DEGs by p-value < 0.05, log_2_FC > 1.5 and log_2_FC < − 1.5. In total, 189 genes were found in P0 mice, and 286 genes were found in adult mice. Thirty genes were found in both P0 and adult synaptic and nonsynaptic region comparisons. Moreover, these DEGs were separated into upregulated and downregulated DEGs. However, no gene was found in the merge of the upregulated P0 and adult mouse synaptic genes. By comparison, 28 genes were downregulated in both the P0 and adult mouse synaptic regions. In addition, comparison of the upregulated genes in P0 mice with downregulated genes in adult mice showed no genes in common. The comparison of the downregulated genes in P0 mice with upregulated genes in adult mice showed 2 genes in common (Fig. 8a). Supposing that the downregulated genes have a negative regulatory function, these results indicate that some genes that exhibit a negative regulatory function in NMJ development may in turn positively regulate NMJ maturation. We next screened the DEGs by p-value < 0.05; log_2_FC > 2 and < − 2. Eleven genes were found to be differentially expressed in both the P0 and adult comparisons (Fig. 8b). All the genes were downregulated in both P0 and adult mice. Eight genes were found to be associated with each other in the PPI network, and these genes were mainly enriched in irregular NMJ functional-related pathways (Fig. 8c, d).

## Discussion

In this study, we identified the DEGs and pathways related to NMJ development, maintenance and maturation. P0 is a stage during which the NMJ is under development. GO enrichment analysis of synaptic and nonsynaptic regions in P0 mice revealed that the DEGs were mainly enriched in chemical synaptic transmission, neuron projection, ion channel activity and neuroactive ligand-receptor interaction. The neuron projection, which is necessary for embryonic development, was identified in GO analysis. In NMJs, normal motor neuron projections are important for NMJ formation and development; specifically, muscle-derived Lrp4 has been proven to be important for presynaptic differentiation during NMJ development [31]. In vitro results also confirmed this finding [32]. The GO results provide us with evidence that these proteins in the neuron projection pathway may regulate presynaptic differentiation, similar to Lrp4. In NMJs, ACh is released from motor neuron terminals and binds to AChRs in postsynaptic muscle. This triggers the opening of ion channels in the muscle, resulting in muscle contraction. AChRs are mainly permeable to sodium and potassium [33]. Coincidently, GO enrichment analysis revealed that proteins were enriched in ion channel activity pathways. This result provides a basis for further study of NMJ transmission-related ion channels. In NMJs, agrin is released from the motor nerve terminal and acts as a ligand for the receptor Lrp4, which is responsible for stimulating AChR clustering. Agrin-Lrp4 signaling is significant for the normal function of NMJs [3, 4]. In our analysis, the genes enriched in the neuroactive ligand-receptor interaction pathway may potentially regulate other unknown pathways in NMJs, this result needs further analysis.

GO enrichment analysis of the highly expressed genes in adult mouse synaptic and nonsynaptic regions revealed that changes in the most significant modules were mainly enriched in myelination and cell adhesion pathways. In the peripheral nervous system, motor neuron axons were wrapped by myelin. Myelin, which is produced by myelinated Schwann cells (MSCs), is protective and ensures electrical transduction by axons [34, 35]. Increasing evidence suggests that MSCs also play an indispensable role in NMJ formation, as ablation of MSCs during NMJ development results in motor axon degeneration without the formation of NMJs [36, 37]. In our results, the myelination pathway was enriched among the synapse-specific DEGs, suggesting that myelination is indispensable for NMJ maintenance.

The analysis of highly expressed genes of synaptic regions in P0 and adult mice revealed enrichment of the mitochondria-associated component pathway. Our findings were consistent with a previous conclusion that mitochondria are abundantly expressed in the postsynaptic specializations of adult skeletal muscle fibers [38]. However, we found that atrophy-related genes (e.g., Trim63 and Fbxo32) were highly expressed in the adult synaptic region compared with the P0 synaptic region. Trim63 and Fbxo32 are two muscle-specific E3 ubiquitin ligases that control the substrate specificity of the proteasome. Both of these proteins contribute to the loss of muscle mass caused by increased protein degradation during muscle atrophy [38, 39], and they are upregulated in muscle atrophy-induced paradigms [40, 41]. This result indicates that during NMJ development, genes associated with protein degradation may be downregulated in synaptic regions. Here, we should note that muscle cells are also contained in the samples, that may result in some genes responsible for muscle maturation will be also enriched in NMJ maturation hub genes. For example, some proteins of Asb family *(Asb2*, *Asb10)* that were previously reported to be highly expressed in muscle are also enriched in NMJ maturation hub genes [42, 43]*.* These Asb proteins were recently reported to play critical role in skeletal myogenesis and development. Asb2β plays a major role in muscle cell proliferation, myoblast fusion and muscle contraction by targeting Notch signaling, Desmin and Filamin A β [44]. While Asb15 was reported to control skeletal muscle development by regulating muscle cell differentiation [45]. However, it is easy to understand that genes responsible for muscle maturation are also critical for NMJ maturation. As the postsynaptic component of NMJ, skeletal muscle maturation is undoubtedly important for NMJ maturation.

## Conclusions

Our present study was designed to identify DEGs involved in NMJ and their related pathways. From the GO and pathway analysis, we found that the neuron projection pathway, ion channel activity and neuroactive ligand-receptor interaction pathway were enriched in the synaptic region of P0 mice, indicating the importance of these functions for NMJ development. In addition, the neuroactive ligand-receptor interaction pathway proteins Ptgir, Gabrb3 and P2rx3 provide references for the study of NMJ and related diseases during NMJ development. The myelination and voltage-gated ion channel activity pathways may be important for NMJ maintenance. Asb family proteins such as Asb2, Asb10 and Asb15 and the proteins Trim63 and Fbxo32 may regulate muscle developmental-related processes. Together, these results provide a foundation for studying NMJ and the pathogenesis of NMJ diseases.

## Data Availability

The data that support the findings of this study are available from the corresponding author upon reasonable request.

## Abbreviations

NMJ: Neuromuscular junction
AChR: Acetylcholine receptor
GO: Gene ontology
KEGG: Kyoto Encyclopedia of Genes and Genomes
DAVID: Database for annotation, visualization and integrated discovery
MSC: Myelinated Schwann cell
RNA-seq: RNA-seq
GSEA: Gene set enrichment analysis
DEGs: Differentially expressed genes
PPI: Protein–protein interaction networks
CMS: Congenital myasthenic syndromes
MG: Myasthenia gravis
ChAT: Choline acetyltransferase
FPKM: Kilobase of transcript per million fragments mapped
P0: Postnatal day 0
FC: Fold change
NF: Neurofilament
SYN: Synapsin
CC: Cellular component
MF: Molecular function
